# Unveiling the Bio-corona Fingerprinting of Potential Anticancer Carbon Nanotubes Coupled with d-Amino Acid Oxidase

**DOI:** 10.1007/s12033-022-00488-y

**Published:** 2022-04-25

**Authors:** Marta Boreggio, Elena Rosini, Cristian Gambarotti, Loredano Pollegioni, Elisa Fasoli

**Affiliations:** 1 Department of Chemistry, Materials and Chemical Engineering “Giulio Natta”, Politecnico di Milano, Piazza Leonardo da Vinci 32, 20133 Milan, Italy; 2 Department of Biotechnology and Life Sciences, University of Insubria, via J.H. Dunant 3, 21100 Varèse, Italy

**Keywords:** d-Amino acid oxidase, Drug delivery, Mass spectrometry, Multi-walled carbon nanotube, Protein corona, Proteomic analysis

## Abstract

The oxidation therapy, based on the controlled production of Reactive Oxygen Species directly into the tumor site, was introduced as alternative antitumor approach. For this purpose, d-amino acid oxidase (DAAO) from the yeast Rhodotorula gracilis, an enzyme able to efficiently catalyze the production of hydrogen peroxide from d-amino acids, was adsorbed onto multi-walled carbon nanotubes (MWCNTs), previously functionalized with polylactic-co-glycolic acid (PLGA) or polyethylene glycol (PEG) at different degrees to reduce their toxicity, to be targeted directly into the tumor. In vitro activity and cytotoxicity assays demonstrated that DAAO-functionalized nanotubes (f-MWCNTs) produced H2O2 and induced toxic effects to selected tumor cell lines. After incubation in human plasma, the protein corona was investigated by SDS-PAGE and mass spectrometry analysis. The enzyme nanocarriers generally seemed to favor their biocompatibility, promoting the interaction with dysopsonins. Despite this, PLGA or high degree of PEGylation promoted the adsorption of immunoglobulins with a possible activation of immune response and this effect was probably due to PLGA hydrophobicity and dimensions and to the production of specific antibodies against PEG. In conclusion, the PEGylated MWCNTs at low degree seemed the most biocompatible nanocarrier for adsorbed DAAO, preserving its anticancer activity and forming a bio-corona able to reduce both defensive responses and blood clearance.

**Supplementary Information:**

The online version contains supplementary material available at 10.1007/s12033-022-00488-y.

## Introduction

The limited efficiency of traditional cancer treatments, chemotherapy and radiotherapy, has required the introduction of new anticancer strategies, like, for example, the oxidation therapy [1, 2]. The Oxidation therapy uses ‘Reactive Oxygen Species’ (ROS), including radicals and non-radicals molecules, such as hydrogen peroxide (H_2_O_2_), which are involved in immune response, in cell signal transduction and in energy production [3, 4], and, when overproduced, can initiate lethal chain reactions that cause oxidative damage. The aim of oxidation therapy is to generate the oxidative stress directly in cancer cells and to selectively kill them [5, 6]. Noteworthy, H_2_O_2_ damages DNA, proteins, and lipids by direct oxidation or via the metal ion Haber–Weiss or Fenton reductions to form reactive hydroxyl radicals, thus enhancing the cytotoxic effect [7]. Considering the limitations of preliminary studies, published in 1950s–1970s, about the injection of hydrogen peroxide into the bloodstream, later efforts were devoted to introduce ROS-producing enzymes, able to induce toxic effects after appropriate stimulation [8]. The enzyme-activated prodrug therapy (EPT) is a two-step antineoplastic strategy designed to specifically deliver a foreign enzyme in malignant cells, which then converts a non-toxic prodrug into a cytotoxic metabolite. In the first step, the enzyme is targeted to the tumor and then an administered prodrug is selectively converted into an active anticancer drug in tumors, to high local concentration [5, 7–9].

Recently, innovative systems, like nanocarriers, characterized by dimensions ranging between 1 and 100 nm have been investigated to improve the efficiency of anticancer drugs delivery [10, 11]. Chemotherapeutic drugs were firstly bound to specific nanostructures and then injected into the blood stream and their internalization into tumor site was obtained by a passive transport through the ‘Enhanced Permeability and Retention” (EPR) effect, due to an abnormal vascular permeability of cancer vessels for the presence of large endothelial pores. Small particles, such as nanocarriers, could extravasate in the interstitial space and accumulate into the tumor site for the inefficient lymphatic drainage [12, 13]. Moreover, appropriate chemical functionalization of nanostructure’s surface could selectively guide the drug’s delivery through active transport [14–16]. Quite recently, many studies reported data concerning animal tests and clinical trials applied to specific functionalized nanoparticles [17, 18].

One type of nanocarriers, studied as potential drug delivery systems, was carbon nanotubes (CNTs), tubular carbon allotropes formed by layers of graphene arranged in two-dimensional hexagonal lattice. There have been different CNTs, based on the number of concentric walls: single-walled (SWCNTs), double-walled (DWCNTs), or multiple-walled (MWCNTs) nanotubes [19, 20]. CNTs seemed to be promising materials to many biomedical applications: scaffold for tissue regeneration, biosensors, and vehicles for different therapeutic molecules (drugs, proteins, antibodies, DNA, enzymes, etc.) [21, 22]. Despite their promising potentiality, many studies have demonstrated CNTs’ toxicity both in cell cultures and in vivo animal models, due to their hydrophobicity and to their low solubility in polar solutions, like in human blood, able to induce aggregation and precipitation [23, 24]. Consequently, the formation of aggregates has determined the activation of different defensive mechanisms, like the immune response and the reticuloendothelial system, causing their elimination from human body [25–29]. Nevertheless, chemical functionalization on CNTs surface could improve biocompatibility and reduce toxicity, influencing interactions with specific physiological proteins and generating the most performing protein corona or bio-corona [30–34]. The protein corona is a layer of physiological proteins formed around CNTs surface, as a consequence of their interaction with biological fluids. The formation of bio-corona is a dynamic process due to “Vroman effect” that involves different forces between nanomaterial and proteins, such as H bonds, Van der Waals forces, π–π stacking binding, and electrostatic and hydrophobic interactions. Protein corona could modify the biological identity of nanomaterials, as its composition could favor the recognition of CNTs as ‘self’-structures, increasing the time of circulation in bloodstream and the achievement of tumor target, and the modulation of immune system, preventing the uptake [35–37]. One of the most studied functionalization methods is the binding of polyethylene glycol (PEG) onto nanoparticles surface. The covalent coupling of PEG chains improves the EPR effect; macromolecules selectively accumulate and remain in solid tumor tissues due to their peculiar pathophysiological characteristics (see above) [8, 9, 12, 38]. Noteworthy, PEG has been demonstrated to improve biocompatibility of CNTs even if there is no comprehensive knowledge about the effects of polymer’s length and PEGylation degree [39–41]. For these reasons, many efforts were devoted to investigate new alternative CNTs’ functionalization processes, able to reduce side effects and toxicity. One example was the coating of CNTs surface with polylactic-co-glycolic acid (PLGA), a FDA-approved polymer for clinical studies. PLGA-MWCNTs showed several advantages, like high transfection rate, reduced toxicity, and a controlled drug release [42]. In fact these functionalizing polymers may both increase the nanoparticles’ circulation time in bloodstream and inhibit macrophages’ functions, so they would seem to favor the exploiting of the EPR effect with the consequent accumulation of nanoparticles in tumor site.

**Fig. 1:**
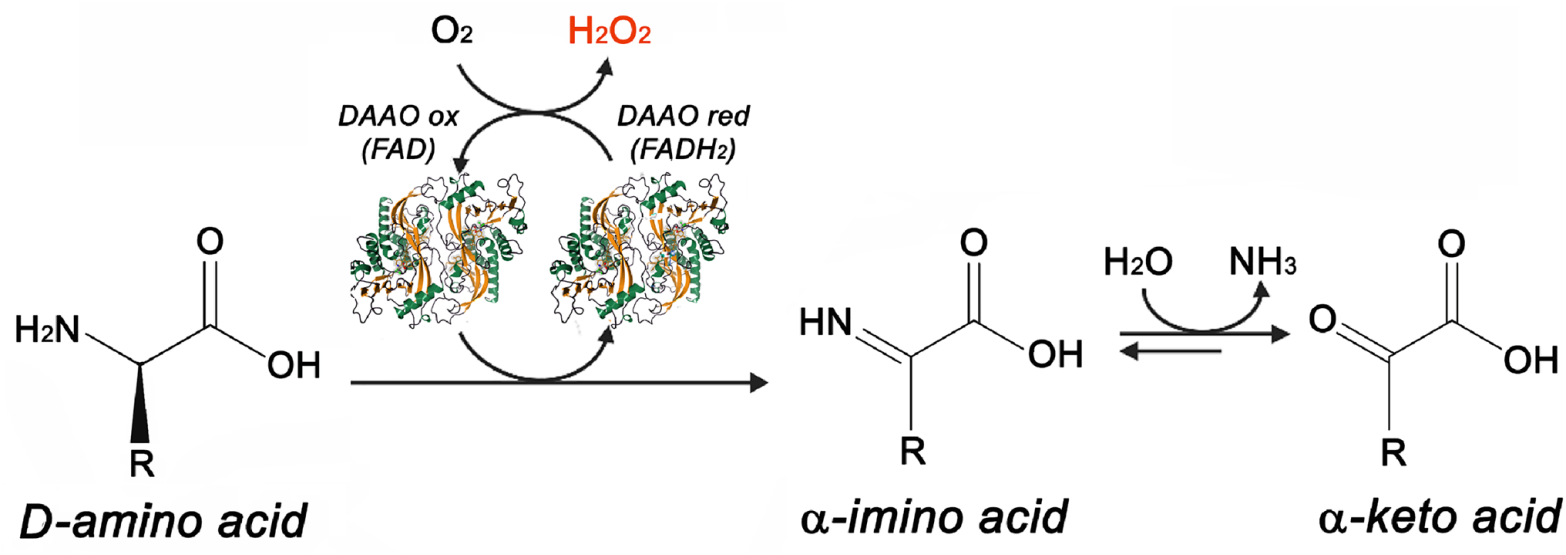
Reaction catalyzed by d-amino acid oxidase: d-amino acids are deaminated into the corresponding α-keto acids, ammonia, and H_2_O_2_ (in red)

Our research aimed to functionalize MWCNTs surface with PEG and PLGA in order to deliver d-amino acid oxidase (DAAO, EC 1.4.3.3) from the yeast *Rhodotorula gracilis* [43, 44], a ROS-producing enzyme, already bound in the past to PEG and nanoparticles [9, 45]. This highly stereoselective flavoenzyme catalyzes the oxidation of d-amino acids into the corresponding α-keto acids, ammonia, and H_2_O_2_ (l-amino acids are neither substrates nor inhibitors, see Fig. 1) [43]. The yeast enzyme was selected because it possesses a very high catalytic activity and a stable interaction with the FAD cofactor [46]. d-Amino acids mainly originate from gut microbiota and foods [47] and in the human body are endogenously present at low concentrations, thus allowing easier regulation of enzyme activity in therapy. Plasma concentrations of d-serine, d-proline, and d-alanine in humans are in the micromolar or submicromolar range [48, 49]. d-Serine and d-aspartate play a crucial role in *N*-methyl-d-aspartate (NMDA) receptor activation and modulation. d-amino acids were recently proposed as useful novel blood-based biomarker for a variety of pathological states [50]. In order to increase d-serine levels in central nervous system as a way to enhance NMDA receptor-mediated neurotransmission, DAAO inhibitors were generated and Luvadaxistat (TAK-831) recently reached phase II of clinical trials to schizophrenia treatment (NCT05182476) [51, 52]. In this work, the DAAO activity was measured by a specific enzymatic assay and the cytotoxicity was demonstrated on different cell lines, like glioblastoma, hepatoblastoma, colon carcinoma, as well as fibroblasts and embryonic cells used as control. Finally, to evaluate the biocompatibility, functionalized MWCNTs (f-MWCNTs) were incubated in human plasma to form the protein corona. The composition of bio-corona was investigated using a proteomic approach based on electrophoretic separation (SDS-PAGE) and mass spectrometry analysis (nLC-MS/MS).

## Materials and Methods

### Synthesis and Carboxylation of MWCNTs

MWCNTs were prepared by the typical Chemical Vapor Deposition (CVD) protocol using acetylene as carbon source [53]. They were then sonicated to reach fragments of an average length of 1–2 µm and subsequently characterized by TEM microscopy, Thermogravimetric Analysis (TGA), Inductively Coupled Plasma (ICP) analysis, and X-ray photoelectron spectroscopy, as reported by Nicoletti et al. [54]. MWCNTs were subsequently oxidized in order to obtain carboxylic functions on their surface using the “Piranha” solution [55]. The carboxylated MWCNTs (MWCNTs-COOH) were then filtered and washed with hot water to a neutral pH of the washing water. Once filtered, MWCNTs were dried at 90 °C overnight.

All the reagents were provided by Sigma-Aldrich® and were used without any further purification.

### MWCNTs Functionalization

MWCNTs-COOH were partially functionalized with PEG 5 kDa or with PLGA 75:25, 66–107 kDa, as shown in Fig. S1. Using the same procedure and different amounts of reagents, two different PEGylation levels (12% and 25%) were obtained. Specifically, 2 separated samples of 200 mg of MWCNTs-COOH were dispersed in 50 mL of dry tetrahydrofuran; then, 100 mg or 500 mg of PEG, 200 mg of *N,N′*-dicyclohexylcarbodiimide, and 60 mg of 4-(dimethylamino)pyridine were added. The two suspensions were sonicated for 2 min and then left under vigorous stirring at room temperature for 6 days, keeping the flask closed in order to avoid evaporation of the solvent. Then, the two mixtures were filtered, washed fives times with 10 mL of hot *N*,*N*-dimethylformamide and five times with 10 mL of boiling methanol, filtered, and then dried overnight at 90 °C.

The MWCNTs functionalization with PLGA was obtained following the procedure reported by Cheng et al., applied on 50 mg of MWCNTs-COOH [42].

All procedures were performed under a fume cover, to avoid vapors toxicity, and wearing a protective mask, to avoid breathing of nanoparticles.

### DAAO Production and Incubation with f-MWCNTs

The recombinant yeast His-DAAO wild type was expressed and purified from BL21(DE3)pLysS *E. coli* cells (Merck Millipore®) as described by Fantinato et al. [56]. The pure enzyme had a specific activity on d-alanine (d-Ala) of 115 U/mg protein at 25 °C, with > 90% purity as judged by SDS-PAGE analysis. The absorbance spectrum of a properly diluted enzyme sample aliquot was used to measure the enzyme concentration (Ɛ_455nm_ = 12.6 mM^−1^ cm^−1^) [46].

A suspension of 2 mg of f-MWCNTs was sonicated in an ultrasound bath for 10 min at room temperature in 400 µL of 50 mM sodium pyrophosphate buffer pH 7.4. The suspension was incubated in 1.5 mL of 2 mg/mL DAAO solution, overnight using a rotating plate at 4 °C with a speed of 15 rpm. The adduct was separated by a magnet from the supernatant, which contains the unreacted DAAO. The supernatant was centrifuged two times at 13,500 rpm (rounds per minute) for 5 min, in order to remove any trace of dispersed nanotubes and the unbound DAAO was determined spectroscopically at 450 nm with a SmartSpec Plus Spectrophotometer (Bio-Rad) on the solution. The amount of protein bound to f-MWCNTs was thus determined as the difference between the starting amount of DAAO and the protein recovered in the supernatant at the end of the reaction.

### DAAO’s Activity and Stability Assay

The activity of the enzyme linked to f-MWCNTs was determined measuring the amount of hydrogen peroxide produced, in the horseradish peroxidase (EC 1.11.1.7; Roche®) and o-dianisidine (Sigma-Aldrich®)-coupled assay using a f-MWCNTs-DAAO’s concentration of 0.26 mg/mL [57].

In order to evaluate the possibility that nanotubes alone may give a signal in the activity assay, the same procedure was applied to f-MWCNTs without DAAO, using a final concentration of 0.2 mg/mL.

The stability was assayed measuring the activity of the enzyme, as described above, every hour for six hours and then overnight. Between successive measurements, samples were maintained under stirring at 37 °C, mimicking the physiological environment.

### Circular Dichroism Spectra

Circular dichroism (CD) spectra measurements were performed to investigate the effect on protein conformation of DAAO adsorption on nanotubes. CD spectra were recorded using a J-810 Jasco spectropolarimeter (Jasco Co., Cremella, Italy) and analyzed by means of Jasco software [58]. The far-UV CD spectrum (190–250 nm) of both native (DAAO) and immobilized (f-MWCNTs-DAAO) enzymes was recorded at 15 °C, with a quartz cuvette (cell pathlength of 0.1 cm). All measurements were performed in 50 mM sodium pyrophosphate buffer, pH 7.4, at 0.1 mg/mL protein concentration and corrected for buffer and f-MWCNTs contributions.

### Cytotoxicity Assay

The cytotoxicity of native DAAO and different f-MWCNT-DAAOs was assessed by the thiazolyl blue tetrazolium bromide (MTT, Sigma-Aldrich®) assay on mouse CT26 (colon carcinoma), human U87 (glioblastoma), and HepG2 (hepatoblastoma) cancer cell lines, as well as on monkey COS-7 (kidney) fibroblasts and human embryonic HEK293 (kidney) cells as control, following the protocol reported by Rosini et al. [59]. Merck Millipore® provided all the cell lines.

Cells plated in 96-well culture plates at a density of 3000 cells per well were cultured overnight at 37 °C in a 5% CO_2_ incubator in Dulbecco’s modified eagle medium (Euroclone), supplemented with 10% fetal bovine serum, 4.5 g/L glucose, 1 mM l-glutamine, 1 mM sodium pyruvate and penicillin–streptomycin. Cells were then exposed to different amounts of enzyme (0.01 or 0.1 U) and D-Ala (1 or 10 or 20 mM) for 24 h. Cytotoxicity assay was performed also on f-MWCNTs without DAAO, as control. Following the removal of the growth medium, 100 μL of 0.5 mg/mL MTT was added; after 4 h at 37 °C, the reagent was removed, 100 μL of dimethylsulfoxide (Sigma-Aldrich®) were added, and the absorbance at 600 nm was recorded. The value measured for the control (i.e., cells incubated without DAAO and/or d‐Ala) was taken as 100% of survival. Toxicity was quantified as the fraction of surviving cells relative to the untreated cells as control. The analyses were replicated five times for each condition and data were analyzed for statistical significance using two-way ANOVA followed by a Tukey’s multiple comparison test using GraphPad Prism software (GraphPad Software Inc., La Jolla, CA). Significance was assessed at *p* < 0.05.

### f-MWCNTs-DAAO’s Incubation with Human Plasma

60 μL of f-MWCNTs-DAAO were mixed with 1940 μL of human plasma (Heat inactivated, from male AB-clotted whole blood, Sigma-Aldrich®) for 4 h at 37 °C. A magnet was used to separate the f-MWCNTs-DAAO-plasma proteins complexes from the human plasma supernatant. The human plasma proteins, linked to MWCNTs-DAAO, were then eluted twice using 30 mM Tris–HCl solution at pH 7.4 (150 and 100 µL, respectively). The detached human plasma proteins were collected for SDS-PAGE analysis. In addition, f-MWCNTs without DAAO were also incubated in human plasma as control, in order to evaluate the affinity of CNTs for human proteins.

### SDS-PAGE

SDS-PAGE gels’ composition was as stated in Nicoletti et al. [54], using 0.02 μg/μL modified porcine trypsin (Thermo®). Particularly*,* discontinuous gels consisted of two parts: a 4% polyacrylamide stacking gel (125 mM Tris–HCl, pH 6.8, 0.1%, m/v, SDS) at the top and a 12% resolving polyacrylamide gel (in 375 mM Tris–HCl, pH 8.8, 0.1%, m/v, SDS buffer). A Tris–glycine buffer at pH 8.3 (with 0.1% SDS, m/v) was employed to fill the cathodic chamber, whereas a Tris buffer at pH 8.8 was used in the anodic chamber.

Carbon nanotubes eluates were treated under both native (solubilized in 2× Laemmli buffer without β-mercaptoethanol at room temperature) and denaturant (solubilized in 2× Laemmli buffer enriched with β-mercaptoethanol at 99 °C for five minutes) conditions and they were loaded onto SDS-PAGE gels as following: 10 μL of 12% PEGylated MWCNTs eluate, 6 μL of 25% PEGylated MWCNTs eluate, and 10 μL of 10% PLGA-MWCNTs eluate, both with and without DAAO.

Electrophoresis was performed with three steps with increasing voltage: first step was set at 50 V for 20 min, second step at 100 V for 40 min, and third step at 150 V until the dye front reached the bottom of the gels. Staining and distaining of gels were performed with Colloidal Coomassie Blue and 7% (v/v) acetic acid in water, respectively.

All protein bands were excited and subjected to washing steps with 50 mM Ammonium Bicarbonate (AmBic) and Acetonitrile (ACN), at 56 °C under stirring, in order to remove all colloidal Blue Coomassie. Afterward, the gel slices were reduced and alkylated with 1.5 mg/mL DTT (in 50 mM AmBic) at 56 °C and 10 mg/mL iodoacetamide (in 50 mM AmBic) at room temperature, respectively. Finally, proteins were digested with 0.02 µg/µL trypsin (in 25 mM AmBic) at 37 °C overnight and cleaned and concentrated with Stage Tips containing reverse phase C_18_ (“GELoader” pipette tip C_18_ material, Thermo Scientific).

### Mass Spectrometry and Data Analysis

8 µl of tryptic-digested samples were injected on a reversed-phase trap column (Acclaim PepMap100, C18, 100 Å, 5 µm, 100 µm ID × 2 cm length, Thermo Scientific) for peptide clean-up and pre-concentration. After clean-up the trap column was placed in series with a fused silica reverse-phase column (PicoFrit column, C18 HALO, 90 Å, 75 µm ID, 2.7 µm, 10.5 cm length, New Objective). A nanochromatography system (UltiMate 3000 RSLCnano System, Thermo Scientific) delivered a constant flow rate of 300 nL/min. The separating gradient ramped linearly from 4% buffer A (2% ACN and 0.1% FA in water) to 96% buffer B (2% water and 0.1% FA in ACN) in 60 min. The eluting peptides were on-line sprayed in a LTQ XL mass spectrometer (Thermo Scientific). Full mass spectra were acquired in the linear ion trap in the mass range *m*/*z* 350 to *m*/*z* 1800 Da. The 5 most intense ions were automatically selected and fragmented in the ion trap. Target ions already selected for fragmentation were dynamically excluded for 30 s.

The MS data were analyzed by the Mascot search engine (Version 2.3.01), using the Proteome Discoverer software (v. 1.2.0 Thermo) and consulting specific UniProtKB/Swiss-Prot protein database (Swiss-Prot_HomoSapiens 1,188,582 sequences and 344,584,622 residues). A preliminary subtraction of common contaminants was performed using definite *Contaminants* database (262 sequence, 133,770 residues). The identified proteins were classified by molecular function using Gene Ontology (GO) analysis (https://www.ebi.ac.uk/QuickGO) and a comparison between all identified plasma proteins was conducted by Venn diagram (https://bioinfogp.cnb.csic.es/tools/venny/).

## Results

### MWCNTs Preparation and Incubation with DAAO

MWCNTs, synthesized by the CVD technique, had external and internal diameters ranging between 14–20 nm and 2–5 nm, respectively, with a mean number of walls of 12–15 and a mean length between 1 and 10 µm. The TGA analysis revealed > 95% w/w graphitic carbon purity and < 5% w/w remaining impurities, due to CVD catalysts (such as Fe and Al) trapped inside the nanotube as confirmed by ICP analysis. MWCNTs were then ultrasonicated to reduce their average length to 1–2 µm and their toxicity, strictly related to dimensions [60, 61]. The first MWCNTs-modification step was the carboxylation, reaching a 4.4% figure as shown by TGA analysis. MWCNTs-COOH were functionalized with PEG (PEG-MWCNTs): two different PEGylation percentages, 12% PEG-MWCNTs and 25% PEG-MWCNTs, were obtained as reported in Fig. S1a and Fig. S2a-b. For what concern the functionalization with PLGA, the TGA has revealed a 10% functionalization (10% PLGA-MWCNTs, Fig. S1b and Fig. S2c).

After incubation of 2 mg of f-MWCNTs with 3 mg of DAAO, the amount of enzyme adsorbed onto nanotubes’ surface was almost the same (~ 0.49 mg) for all f-MWCNTs.

As shown in Fig. S3, the far-CD spectra (related to the secondary structure content) of f-MWCNTs-DAAO samples were superimposable and significantly different from the one for the free enzyme; the different functionalization of MWCNTs, as well as the different PEGylation percentages used, altered the DAAO conformation to a similar extent. The alteration in flavoenzyme conformation agrees with the decrease in enzymatic activity detected upon enzyme adsorption (see below).

### DAAO Activity and Stability

**Fig. 2:**
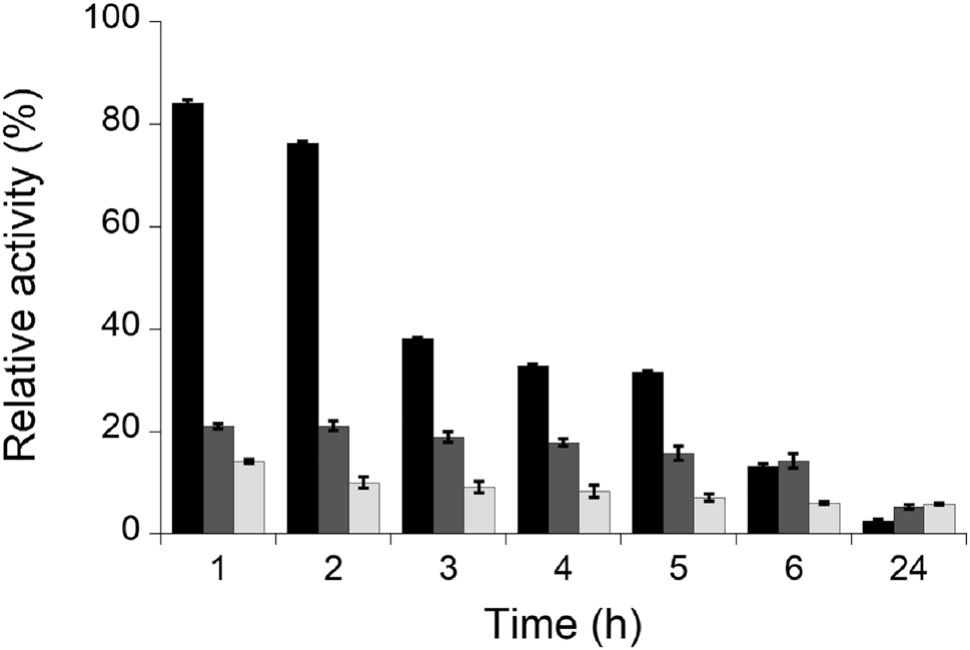
Enzyme stability at 37 °C. The assay was performed on free DAAO (black bars), 12% PEG-MWCNTs-DAAO (dark gray bars), and 10% PLGA-MWCNTs-DAAO (light gray bars). The activity value at 0 h for each sample is taken as 100%. The values are reported as mean ± standard deviation (*n* = 3)

The enzymatic activity of DAAO linked to all f-MWCNTs was lower than the value obtained for the free enzyme; a figure of 19, 24, and 76 U/mg was apparent for 12% PEG-MWCNTs-DAAO, 10% PLGA-MWCNTs-DAAO, and free DAAO, respectively. Concerning the enzymatic stability, the free enzyme showed a linear decrease of activity up to 2 U/mg after overnight incubation at 37 °C (i.e., 2.6% of the initial activity). For the f-MWCNTs-DAAO samples, a similar activity was recorded after 24 h. This value corresponds to 6% of the initial activity, a figure higher than for free DAAO (Fig. 2). No data are available at 25% PEG since the increase of PEGylation strongly reduced the enzymatic activity. The f-MWCNTs alone (without DAAO) showed no activity.

### Incubation of f-MWCNTs and f-MWCNTs-DAAO with Human Plasma

After incubation with human plasma, SDS-PAGE analysis of the proteins interacting with DAAO-nanotubes has depicted similar profiles for all bio-corona formed onto PEGylated MWCNTs (Fig. S4a–b**)**, even if the increase of functionalization percentage resulted in an enhanced intensity of bands. Moreover, bio-corona of PLGA-nanotubes was characterized by more numerous and more intense bands than the PEGylated ones (Fig. S4c).

**Fig. 3:**
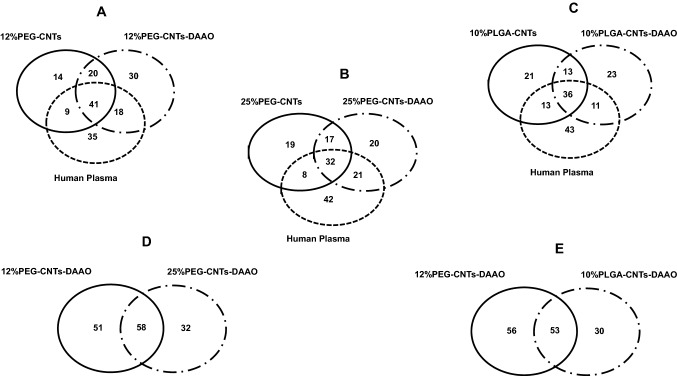
Venn diagrams report proteins belonging to bio-corona of PEGylated and PLGA nanotubes, with and without DAAO. **A** compares proteins captured by 12% PEG-MWCNTs, 12% PEG-MWCNTs-DAAO, and those identified in human plasma, **B** shows proteins on 25% PEG-MWCNTs, on 25% PEG-MWCNTs-DAAO, and those identified in human plasma, **C** reports proteins captured by 10% PLGA-MWCNTs, by 10% PLGA-MWCNTs-DAAO, and those identified in human plasma, **D** and** E** compare proteins captured by 12% PEG-MWCNTs-DAAO/PEG-MWCNTs-DAAO and 12% PEG-MWCNTs-DAAO/10% PLGA-MWCNTs-DAAO, respectively

After mass spectrometry analysis (Supplementary Tables), all identifications were compared by Venn diagrams. PEGylated nanotubes with DAAO have interacted with more proteins than the same nanostructures without the enzyme: 109 vs 84 for 12% PEGylated samples and 90 vs 76 for 25% PEGylation (Fig. 3a, b). Considering MWCNTs functionalized with PLGA, protein analysis demonstrated no significant differences in number of identified proteins related with DAAO presence (Fig. 3c). Furthermore, the high percentage of PEGylation or the presence of PLGA reduced the number of identifications: 109 vs 90 for 12% and 25% PEGylated nanotubes and 109 vs 83 for MWCNTs functionalized with 12% PEG and PLGA (Fig. 3d, e).

**Fig. 4:**
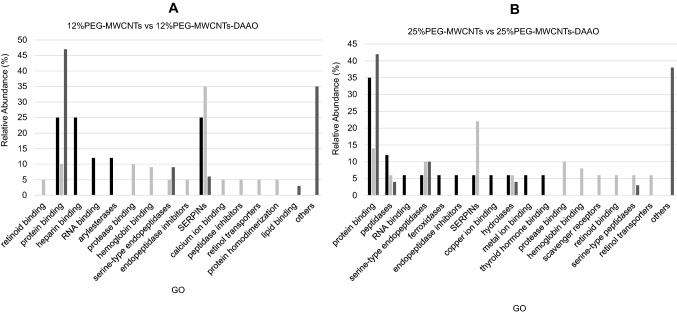
GO analysis of proteins found in bio-corona of 12% and 25% PEGylated nanotubes. **A** Histograms compare GO functions, specifically found in 12% PEG-MWCNTs (black bars) and 12% PEG-MWCNTs-DAAO (light gray bars) or recognized commonly (dark gray bars). **B** Histograms compare GO functions, specifically found in 25% PEG-MWCNTs (black bars) and 25% PEG-MWCNTs-DAAO (light gray bars) or recognized commonly (dark gray bars). SERPINs are serine-type endopeptidases inhibitors, while Retinol transporters are retinol transmembrane transporters. The GO class ‘others’ is referred to chaperone binding, calcium-dependent protein binding, antigen binding, signaling receptor binding, and enzyme binding

A GO analysis of proteins’ molecular functions was performed to investigate the involvement of specific biological processes. Distinct protein classes were specifically found in different MWCNTs’ functionalization; the increase of PEGylation percentage seemed to favor the selective binding of scavenger receptor proteins, present on macrophages cells, and to reduce the interaction with lipid binders, possible dysopsonins (Fig. 4).

**Fig. 5:**
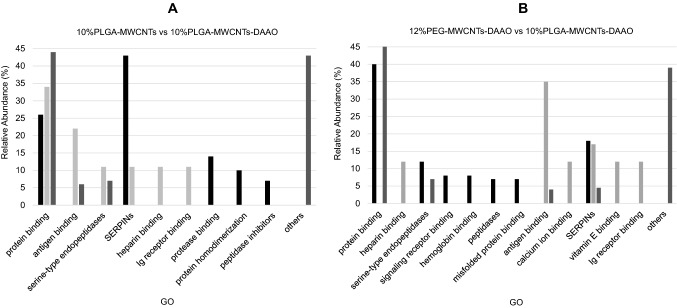
GO analysis of proteins found in bio-corona of 10%PLGA nanotubes. **A** Histograms compare GO functions, specifically found in 10% PLGA-MWCNTs (black bars) and 10% PLGA-MWCNTs-DAAO (light gray bars) or recognized commonly (dark gray bars). **B** Histograms compare GO functions, specifically found in 12% PEG-MWCNTs-DAAO (black bars) and 10% PLGA-MWCNTs-DAAO (light gray bars) or recognized commonly (dark gray bars). GO group identified as ‘others’ refers to chaperone binding, calcium-dependent protein binding, lipid binding, arylesterase activity, and enzyme binding. SERPINs are serine-type endopeptidases inhibitors. ‘Ig’ stands for immunoglobulin

Instead, PLGA functionalization seemed to promote the interaction with immunoglobulin receptor binders, able to activate both the immune and inflammatory responses (Fig. 5). Also the presence of DAAO in PEGylated nanotubes seemed to favor the interaction with serine-type endopeptidase inhibitor proteins (Serpins), serine-type endopeptidases, and retinoid binders.

### Cytotoxicity Assay

**Fig. 6:**
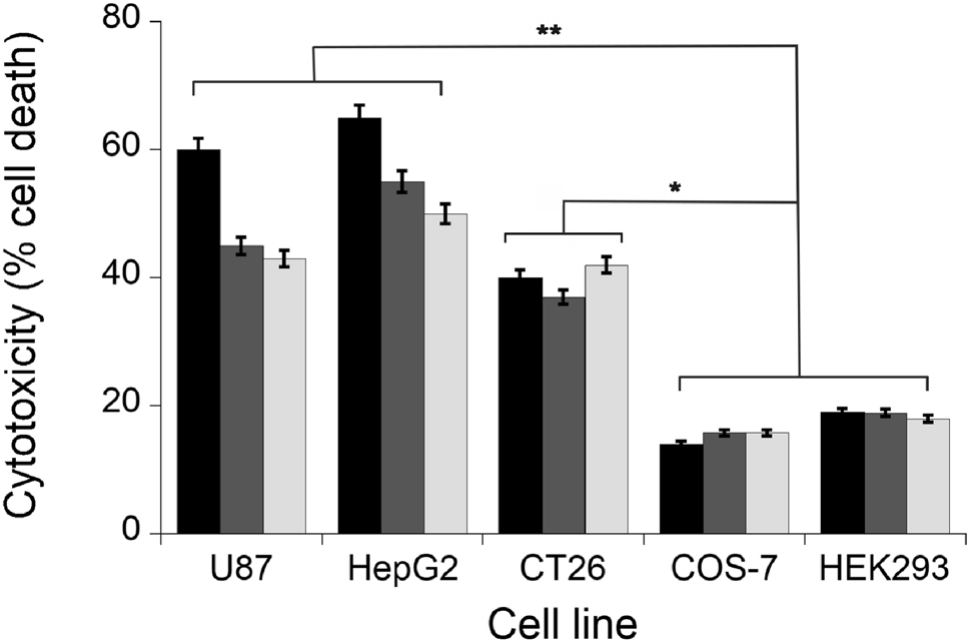
Cytotoxicity induced by DAAO treatment. Cytotoxicity of free DAAO (black bars), 12% PEG-MWCNTs-DAAO (dark gray bars), and 10% PLGA-MWCNTs-DAAO (light gray bars), on the indicated tumor cell lines and comparison with the control cell lines (COS-7 and HEK293), using 0.01 U of enzyme and 20 mM of D-Ala [59]. Toxicity was quantified as the fraction of surviving cells relative to the untreated ones as control (i.e., the cells incubated without DAAO or D-Ala) taken as 100% of survival. The values are reported as mean ± standard deviation (*n* = 5). The results were evaluated by statistical analysis using two-way ANOVA followed by a Tukey’s multiple comparison test. **p < 0.0001, *p < 0.001

In vitro cytotoxicity assay was performed on human and monkey tumor (CT26, U87, HepG2) and control (COS-7 and HEK293) cell lines, using d-Ala as the optimal substrate, and the same enzymatic units (0.01 U). This effect was significantly more evident on tumor cells in comparison to control cells (i.e., COS-7 fibroblasts and HEK293 embryonic cell lines) (Fig. 6). A remarkable and similar cytotoxicity was apparent for both 12% PEG-MWCNTs-DAAO and 10% PLGA-MWCNTs-DAAO, showing a dependence on d-Ala concentration (Fig. S5), thus pointing to a strict control of cytotoxicity in vivo from exogenous D-amino acid supply. In all experiments, 0.044 mg and 0.03 mg of nanotubes functionalized with PEG and PLGA, respectively, were employed and the use of higher amounts of CNTs was hampered by interferences with plate’s surface. Both MWCNTs-COOH and functionalized (12% PEG-MWCNTs and 10% PLGA-MWCNTs) nanotubes did not induce cytotoxicity.

## Discussion

The attempt to design a biocompatible drug delivery system was performed by functionalizing MWCNTs with PEG and PLGA to improve the solubility of nanostructures. PEG was chosen considering the large biomedical applications of 5 kDa PEG, exploiting its biocompatibility and its capability to increase the circulation time of CNTs in the bloodstream and to preserve the cytotoxic effect of DAAO [9, 62, 63]. Two different PEGylation percentages were tested: 12% because it was in the typical range used in literature (10–20%) and 25% to increase the adsorption of DAAO molecules onto nanotubes’ surface. Despite the high percentage of PEGylation, no significant differences of adsorbed DAAO were apparent.

In order to deeply investigate the possible application of our nanocarriers, we evaluated the activity, the stability, and the cytotoxicity of immobilized DAAO, comparing the data with those of free enzyme. First of all, DAAO adsorbed onto f-MWCNTs showed a lower activity than free DAAO, probably due to the interaction with CNTs and/or with functionalizing polymers. Actually, the enzyme immobilization onto nanocarriers could involve residues close to the active site or to the FAD binding site, thus inducing a conformational change affecting the biological function, as confirmed by the changes observed in CD spectra reported in Fig. S3. The enzymatic activity of 25%PEG-MWCNTs-DAAO was so low to prevent any further assay.

As reported in literature, DAAO activity showed a time-dependent decrease reaching a full inactivation after 24 h at 37 °C [43, 64]. The enzyme inactivation was slower for DAAO present onto CNTs. The cytotoxicity of tested functionalized nanotubes depends on the concentration of the administered substrate (D-Ala, the prodrug substrate), as shown in Fig. S5 [45]. Cytotoxicity assays have compared the effects of both free enzyme and f-MWCNTs-DAAO on cancer cell lines vs. COS-7 and HEK293 cell lines, used as control. All DAAO forms used showed a cytotoxicity most evident on tumor cells as compared with COS-7 fibroblasts or HEK293 embryonic control cells (Fig. 6) [7]. The adsorbed DAAO gave a percentage of cell death on mouse colon carcinoma CT26 cell line comparable to free DAAO, while the human U87 and HepG2 tumor cells were more affected by free DAAO (60–65% cell death) compared to adsorbed DAAO (45–55% cytotoxicity) (Fig. 6) [59]. Antioxidative enzymes such as catalase, superoxide dismutase, and glutathione peroxidase are expressed at a lower extent in tumor cells in comparison to normal cells, thus increasing tumor vulnerability to ROS [65, 66]. The H_2_O_2_ produced in the blood stream by circulating MWCNTs-DAAO does not induce significant side effects since a 50-fold higher catalase activity in blood compared to tumor tissues was reported [66]. Consequently, the production of H_2_O_2_ by MWCNTs-DAAO regulated by d-alanine administration will be attained without any toxicity to normal tissues.

We next investigated the biocompatibility of functionalized nanocarriers strictly connected with the composition of protein shell on carbon nanotubes’ surface. Actually, protein corona is responsible for the biological fate of nanostructures because it could favor blood circulation for extended time, the targeting to the tumor site, and to evade clearance by the immune system [67]. The indispensable “stealth effect” of nanocarrier could be pursued with a balance between the thickness and the composition of protein corona (i.e., an enrichment of anti-inflammatory proteins). The adsorption capacity is dependent on the physicochemical properties of nanostructures and of functionalizing groups; on this side, PEGylation reduces protein adsorption, suppressing any non-specific protein interaction and PLGA is a promising polymer for its biodegradability and biocompatibility [68–71].

Mass spectrometry analysis of protein corona generated on DAAO nanotubes surface, after human plasma incubation, confirmed the relationship between bio-corona’s thickness and composition. A higher number of adsorbed proteins were identified onto PEGylated CNTs: 109 for 12% PEG-MWCNTs vs. 83 for 10% PLGA-MWCNTs. The presence of the enzyme onto PEGylated CNTs has favored the adsorption of proteins, increasing the number of identifications in bio-corona: 84 vs. 109 for 12% PEG-MWCNTs and 76 vs. 90 for 25% PEG-MWCNTs. This was not observed with PLGA, probably for the major steric hindrance of polymer compared to DAAO.

Considering the composition of protein corona and the data reported in Supplementary Tables, positive acute-phase proteins and immunoglobulins (Igs) were commonly found in all bio-corona, denoting their role in inflammation and immune response [72, 73]. Acute-phase proteins are normally present in human plasma at basal concentration, where mediate the inflammatory process by preventing tissue damage. Some acute-phase proteins, like prothrombin, fibrinogen alpha chain, and coagulation factor XII, identified in all f-MWCNTs, promote the complement’s activation and the coagulation process, increasing their level in bloodstream [74, 75]. On the contrary, the high concentration in human plasma of other acute-phase proteins, like α-2-macroglobulin present on the surface of both PEGylated and PLGA-nanotubes, inhibits the lectine pathway, controlling the complement’s cascade [76–81]. Accordingly, all f-MWCNTs have adsorbed more cascade’s activators, like complement factor B1 and complement C1s, than inhibitors, such as C4-binding protein alpha chain [82, 83]. Some identified proteins were involved in the upregulation of Igs’ production, like retinol-binding protein 4, or were linked with the activation of macrophages’ anti-inflammatory pattern, such as CD5 antigen like [84]. All these proteins, together with Igs, are defined opsonizing because enhance the macrophages uptake. Only by eluding phagocytic cells, nanocarriers reach their destination within the body and their presence may lead to a reduction of biocompatibility.

The functionalization and drug’s influence on bio-corona composition were highlighted also by our results. Compared to PEG-DAAO nanotubes, the protein corona of PLGA-MWCNTs-DAAO showed an increase of opsonizing proteins (such as Igs, immunoglobulin receptor binders, and hemopexin, involved in the upregulation of Igs’ synthesis) that could contribute to nanocarriers deactivation by macrophages uptake. The putative intense activation of immune response associated to 10% PLGA-MWCNTs-DAAO could be due to both the polymer’s hydrophobicity and the nanocarrier’s dimensions [85, 86]. A similar result was apparent increasing the PEGylation percentage; the bio-corona of 25% PEGylated CNTs triggered immune and inflammatory response activation, interacting with more Igs and scavenger receptor proteins (able to enhance the macrophages uptake and participating to the innate immune recognition) [87, 88]. Indeed, the identification of many Igs has confirmed previous data that PEG may cause hypersensitivity reactions due to antibodies formation, which can lead to complement activation and an accelerated blood clearance [89, 90].

In all PEGylated nanotubes, the presence of DAAO has enriched the bio-corona of positive acute-phase proteins, like alpha-2-antiplasmin and haptoglobin-related protein. Instead, in both 12% PEG-MWCNTs and 10% PLGA-MWCNTs many apolipoproteins were identified mostly after enzyme adsorption. Apolipoproteins are well-known proteins to be responsible of the fate of nanocarriers (i.e., by promoting the passage through blood–brain barrier and the transport into central nervous system) [91, 92]. These proteins have been defined as dysopsonins, able to minimize the macrophages uptake and the clearance from the bloodstream, and they contribute to stealth nanostructures [93].

Our results identified 12% PEG-MWCNTs as the most biocompatible f-MWCNTs among all tested ones, because of a balance between thickness and advantageous biological properties of bio-corona. Moreover, they adsorbed the therapeutic enzyme preserving part of its activity and showing an increased stability and a good cytotoxicity on tested tumor cell lines. This work could represent a starting point for future nanocarrier design which may evaluate not only the type of functionalization or polymer used to coat nanostructures, but also the affinity for biological proteins present in human fluids, responsible for bio-corona formation and for stealth effect.

## Conclusion

The present research has functionalized MWCNTs with PEG, the most currently used polymer in biomedical field of drug delivery, and with PLGA, a biodegradable polymer, to generate a promising candidate for a selective antitumor therapy based on in situ generation of ROS controlled by d-alanine administration (“activity on demand”), an approach that seems well suited to now be evaluated in vivo [9, 94]. We confirmed that immunogenicity of PEG is highly dependent on the degree of PEGylation and protein corona around 25% PEG-MWCNTs seemed to mostly enhance the immune response, the inflammation, and the macrophages uptake. Despite the preservation of DAAO activity and cytotoxicity, 10% PLGA-MWCNTs had a major affinity than 25%-PEGylated ones for opsonins, favoring the blood clearance. The 12% PEG-MWCNTs were identified as the most promising biocompatible nanocarriers for DAAO because, in addition to maintaining the cytotoxic effects of the enzyme, their bio-corona was enriched in apolipoproteins and anti-inflammatory proteins. Therefore, PEG seemed to be the most efficient polymer also to functionalize MWCNTs for biomedical applications.

## Supplementary Information

Below is the link to the electronic supplementary material.

Supplementary file1 (PPTX 1080 kb)

Supplementary file2 (XLSX 523 kb)
