# Supplementary material for: Unveiling the Bio-corona Fingerprinting of Potential Anticancer Carbon Nanotubes Coupled with d-Amino Acid Oxidase
Source: Mol Biotechnol. 2022 Apr 25;64(10):1164–76. doi: 10.1007/s12033-022-00488-y (PMC9411096; doi:10.1007/s12033-022-00488-y)
Supplement: Supplementary file 1 — Supplementary file1 (PPTX 1080 kb) [file 12033_2022_488_MOESM1_ESM.pptx]

## Slide 1
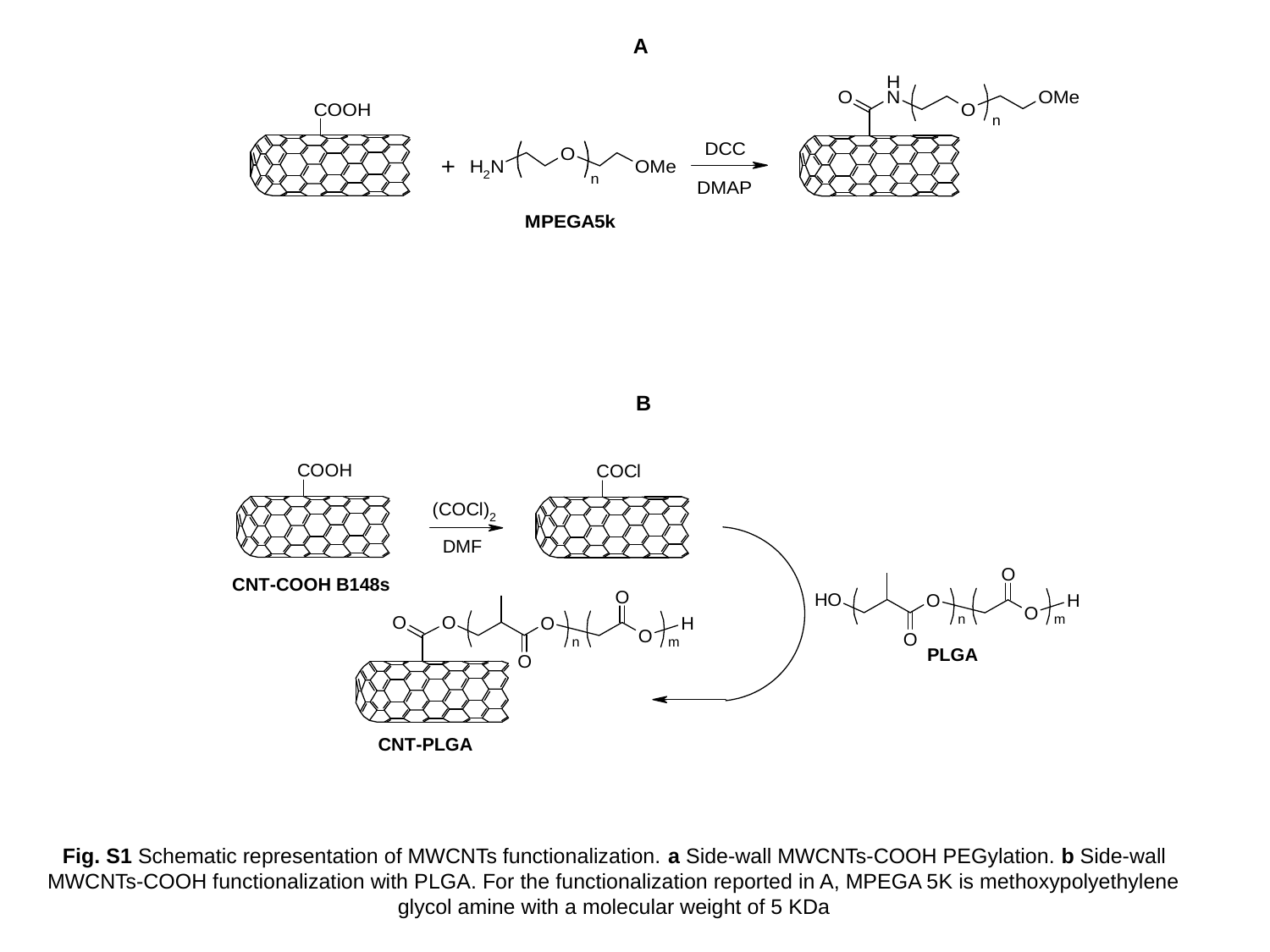

A
B
Fig. S1 Schematic representation of MWCNTs functionalization. a Side-wall MWCNTs-COOH PEGylation. b Side-wall MWCNTs-COOH functionalization with PLGA. For the functionalization reported in A, MPEGA 5K is methoxypolyethylene glycol amine with a molecular weight of 5 KDa

## Slide 2
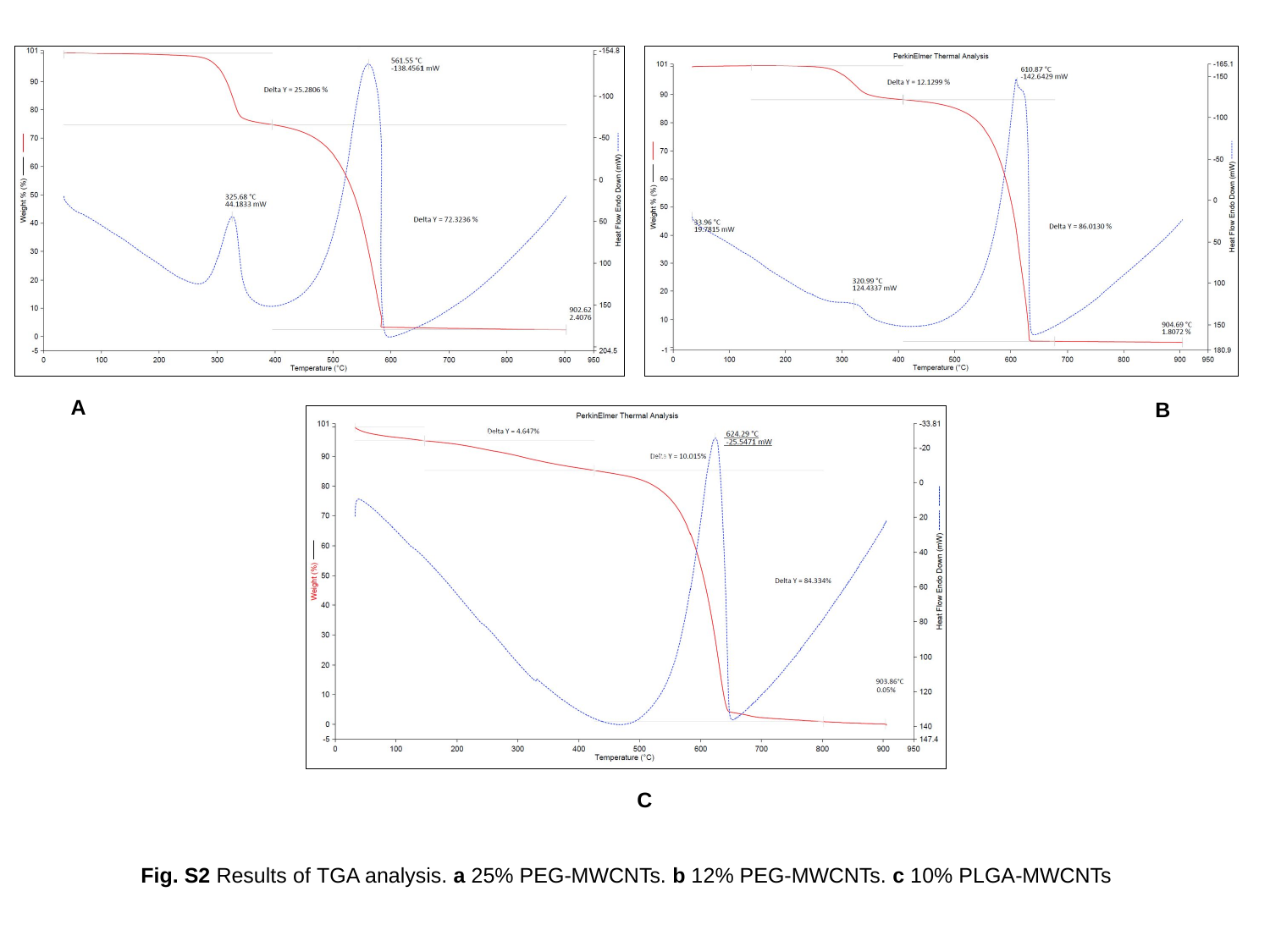

A
B
C
Fig. S2 Results of TGA analysis. a 25% PEG-MWCNTs. b 12% PEG-MWCNTs. c 10% PLGA-MWCNTs

## Slide 3
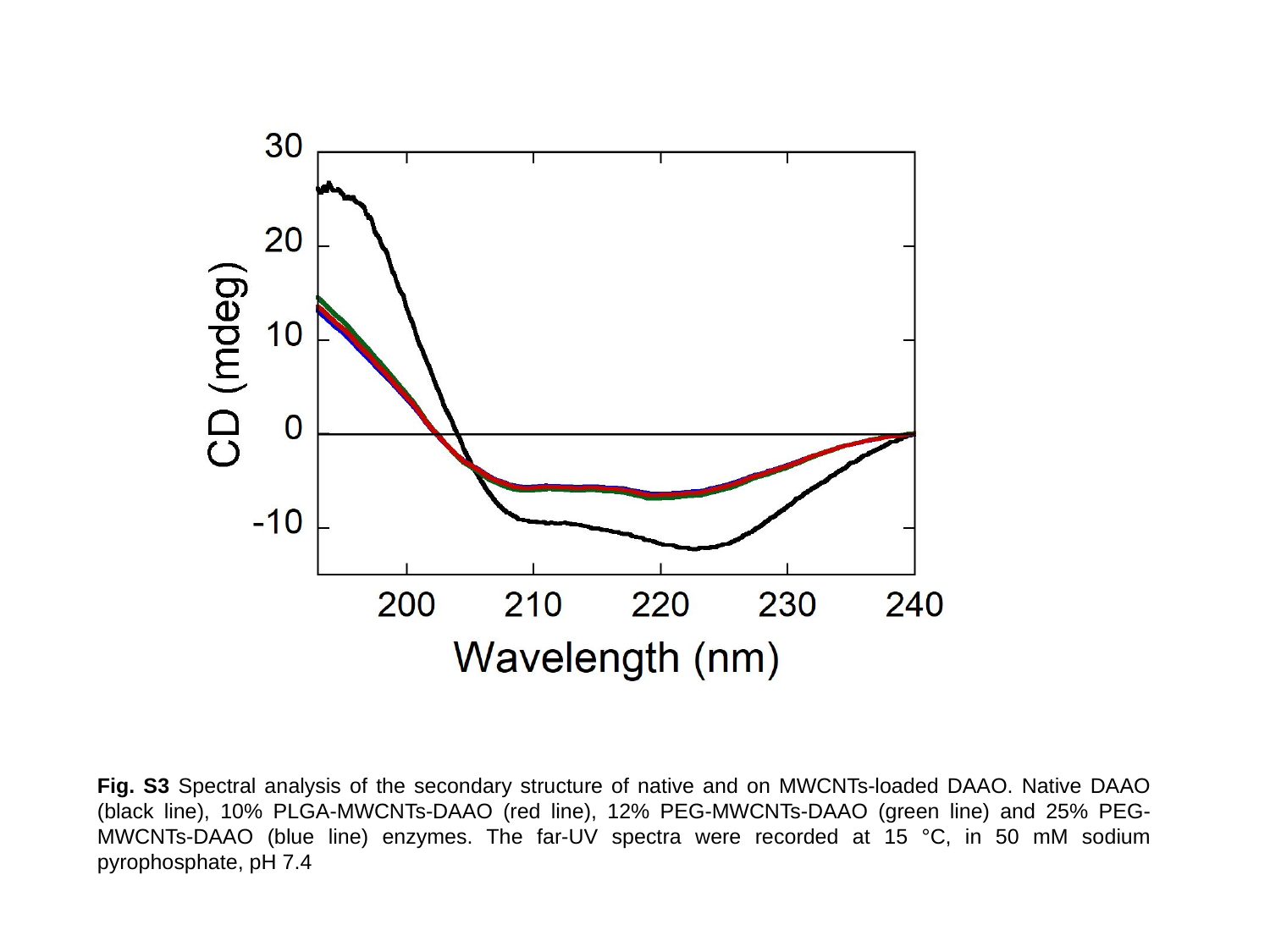

Fig. S3 Spectral analysis of the secondary structure of native and on MWCNTs-loaded DAAO. Native DAAO (black line), 10% PLGA-MWCNTs-DAAO (red line), 12% PEG-MWCNTs-DAAO (green line) and 25% PEG-MWCNTs-DAAO (blue line) enzymes. The far-UV spectra were recorded at 15 °C, in 50 mM sodium pyrophosphate, pH 7.4

## Slide 4
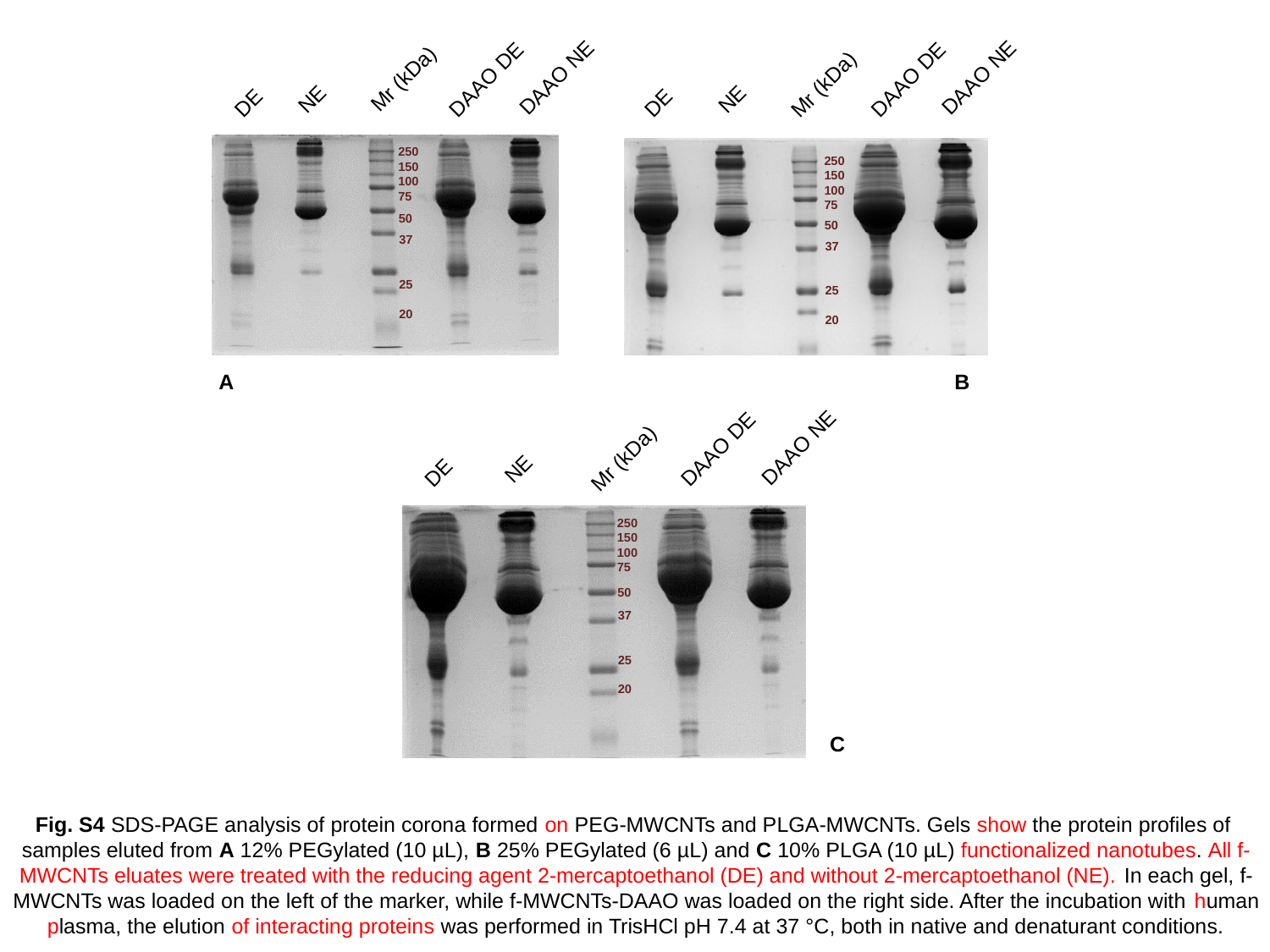

DAAO NE
DAAO NE
Mr (kDa)
DAAO DE
DAAO DE
Mr (kDa)
NE
NE
DE
DE
250
150
100
75
250
150
100
75
50
50
37
25
20
37
25
20
A
B
DAAO NE
DAAO DE
Mr (kDa)
NE
DE
250
150
100
75
50
37
25
20
C
Fig. S4 SDS-PAGE analysis of protein corona formed on PEG-MWCNTs and PLGA-MWCNTs. Gels show the protein profiles of samples eluted from A 12% PEGylated (10 µL), B 25% PEGylated (6 µL) and C 10% PLGA (10 µL) functionalized nanotubes. All f-MWCNTs eluates were treated with the reducing agent 2-mercaptoethanol (DE) and without 2-mercaptoethanol (NE). In each gel, f-MWCNTs was loaded on the left of the marker, while f-MWCNTs-DAAO was loaded on the right side. After the incubation with human plasma, the elution of interacting proteins was performed in TrisHCl pH 7.4 at 37 °C, both in native and denaturant conditions.

## Slide 5
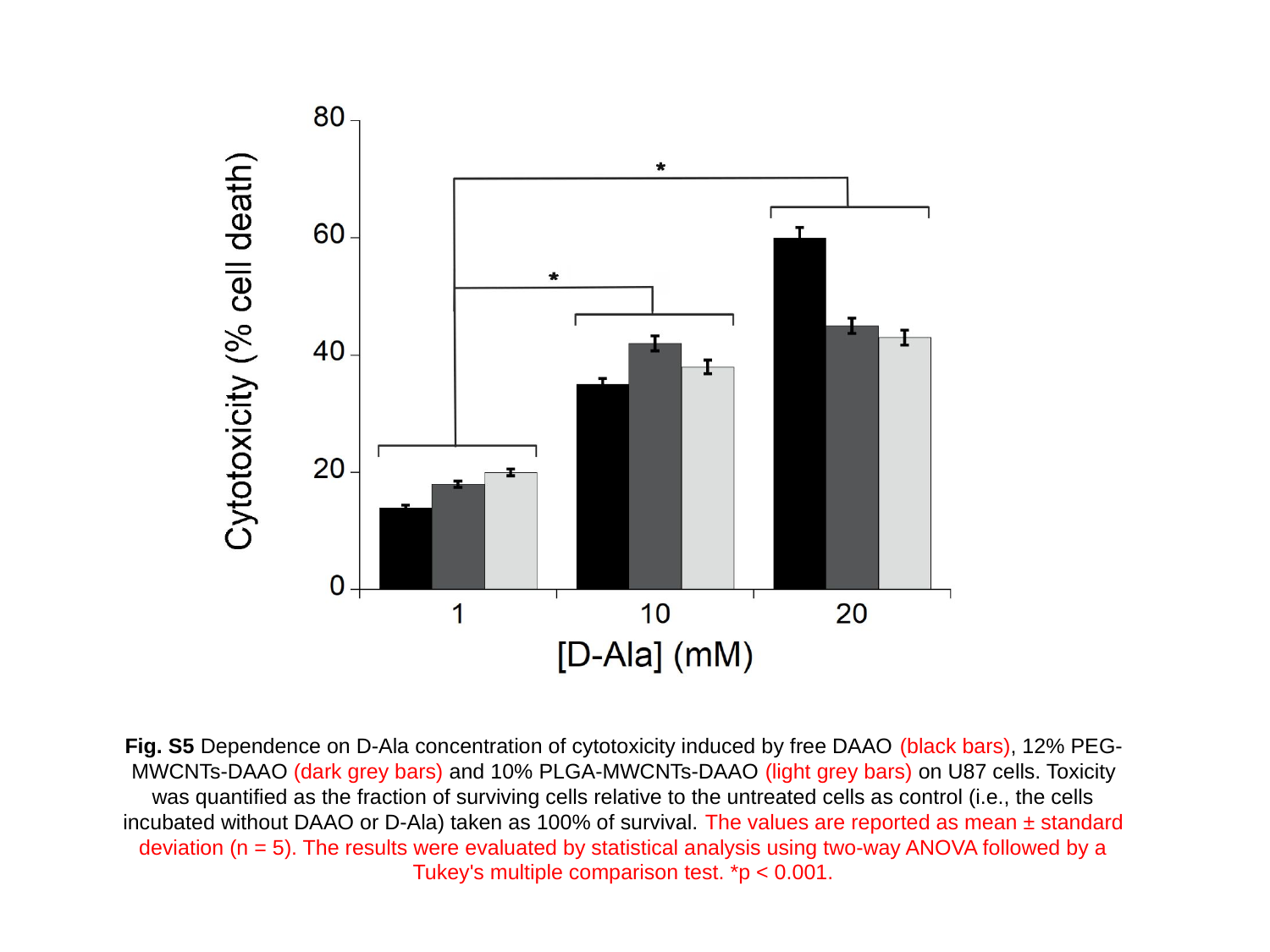

Fig. S5 Dependence on D-Ala concentration of cytotoxicity induced by free DAAO (black bars), 12% PEG-MWCNTs-DAAO (dark grey bars) and 10% PLGA-MWCNTs-DAAO (light grey bars) on U87 cells. Toxicity was quantified as the fraction of surviving cells relative to the untreated cells as control (i.e., the cells incubated without DAAO or D-Ala) taken as 100% of survival. The values are reported as mean ± standard deviation (n = 5). The results were evaluated by statistical analysis using two‐way ANOVA followed by a Tukey's multiple comparison test. *p < 0.001.
